# Integrating geospatial, hydrogeological, and geophysical data to identify groundwater recharge potential zones in the Sulaymaniyah basin, NE of Iraq

**DOI:** 10.1038/s41598-025-94603-z

**Published:** 2025-03-22

**Authors:** Sarkhel H. Mohammed, Musaab A. A. Mohammed, Hawber Ata Karim, Diary A. Mohammed AL-Manmi, Bakhtiar Qader Aziz, Asaad I. Mustafa, Péter Szűcs

**Affiliations:** 1 Institute of Water Resources and Environmental Management, University of Miskolc, Miskolc, Hungary; 2 Department of Earth Sciences and Petroleum, College of Science, University of Sulaimani, Sulaymaniyah, Iraq; 3 Technical College of Engineering, Sulaimani Polytechnic University, Sulaymaniyah, Iraq; 4 National Laboratory for Water Science and Water Security, Institute of Water Resources and Environmental Management, University of Miskolc, Miskolc, Hungary

**Keywords:** Electrical resistivity tomography, Geospatial modeling, Groundwater potential, Multi-criteria, Water resources management, Climate sciences, Environmental sciences, Hydrology

## Abstract

Groundwater is a critical resource for sustaining human activities, particularly in urban areas, where its importance is exaggerated by growing water demands, urban expansion, and industrial activities. Ensuring future water security necessitates an in-depth understanding of groundwater recharge dynamics, which are often complex and influenced by rapid urbanization. The alarming decline in groundwater resources in both urban and rural regions underscore the urgency for advanced groundwater management strategies. However, identifying and evaluating groundwater recharge potential zones (GWPZs) remains a challenge due to the dynamic interplay of hydrogeological and urban development factors. This study employs an integrated approach combining geographic information system (GIS), remote sensing, and multi-criteria decision analysis using the analytical hierarchy process (MCDA-AHP) to delineate GWPZs in the Sulaymaniyah Basin (SB). The methodology is further supported by hydrogeological data and validated through geophysical investigation using electrical resistivity tomography (ERT) data. For the MCDA-AHP, six thematic layers including rainfall, geology, lineament density, slope, drainage density, and land use/land cover were derived from satellite imagery, geological surveys, and well data. These layers were ranked based on their relative influence on groundwater recharge and integrated using GIS-based weighted overlay analysis to generate groundwater potential maps. The results identified three potential zones for groundwater recharge: low (11.26%), moderate (45.51%), and high (43.23%). Validation using ERT data and receiver operating characteristics (ROC) analysis revealed strong agreement, with an area under the curve (AUC) accuracy of 86%. These findings demonstrate the robustness of the integrated approach, providing a reliable tool for minimizing hydrogeophysical exploration costs and reducing the number of unsuccessful boreholes.

## Introduction

The increasing water demand due to the expansion of agriculture and the ever-growing population increases the need for extensive exploration for groundwater resources^1–4^. Due to the changes in climate and low annual distribution of rainfall, the replenishment of groundwater is inadequate. Therefore, one of the most important approaches is to analyze the potential zones for groundwater. In earlier decades, groundwater mapping relied on extensive field surveys and drilling techniques, which were accurate but costly and time-consuming. To reduce these challenges, multi-criteria decision analysis, based on remote sensing data and geographical information systems (GIS), provides a systematic framework for integrating various factors influencing groundwater potential. Remote sensing offers up-to-date, large-scale data on critical parameters such as land use, topography, and hydrological features. GIS support spatial analysis and the integration of these data layers. Together, multi-criteria decision analysis helps to identify and prioritize groundwater recharge zones more efficiently and at a lower cost, making groundwater exploration faster and more sustainable in data scarce region, inaccessible areas and urban area.

In recent decades, the MCDA-based analytical hierarchy process (AHP), developed by^5^ has been conducted and appeared in numerous studies^6–10^. These studies indicated that this technique is capable of being reliable to mapping groundwater potential zones. Although the AHP has proven to be a successful method for groundwater potential mapping and recharge zone identification, many studies fall short in providing a reliable validation of their results. In most cases, validation is performed using indicators such as groundwater flow or depth of the boreholes, which, while informative, fail to account for subsurface complexities and can lead to unreliable conclusions. On the other hand, geophysical methods can play a successful role in validating groundwater potential models derived from the AHP^11–13^. These methods provide information that surface-based indicators cannot, offering a more reliable assessment of groundwater potential zones. Among these techniques, electrical resistivity tomography ERT is particularly effective due to its ability to produce high-resolution images of subsurface resistivity distributions. This method is especially useful for identifying hydrogeological units, determining aquifer boundaries, and understanding variations in hydrogeological properties such as porosity and permeability. The integration of geospatial and geophysical data can verify subsurface conditions such as aquifer boundaries, lithological variations, and hydrogeological properties, enhancing the credibility of AHP-based models and supports more sustainable water resource management practices^3,12,14,15^.

The Sulaymaniyah Basin (SB) faces significant challenges due to the rising demand for groundwater, primarily driven by population growth and urbanization. Consequently, the region requires a detailed assessment of its groundwater recharge potential. Recently, there have been alarming declines in groundwater levels and overexploitation of aquifers, raising serious concerns. To address this issue, our study aims to identify groundwater potential zones (GPWZs) using the multi-criteria decision analysis and AHP approach, supported by geophysical and hydrogeological data, to produce comprehensive GWPZ mapping. Unlike previous studies that often rely on surface indicators for validation, this integrated geospatial-geophysical approach enhances the reliability of groundwater potential mapping by coupling surface and subsurface data.

## Materials and methods

### Study area

**Fig. 1:**
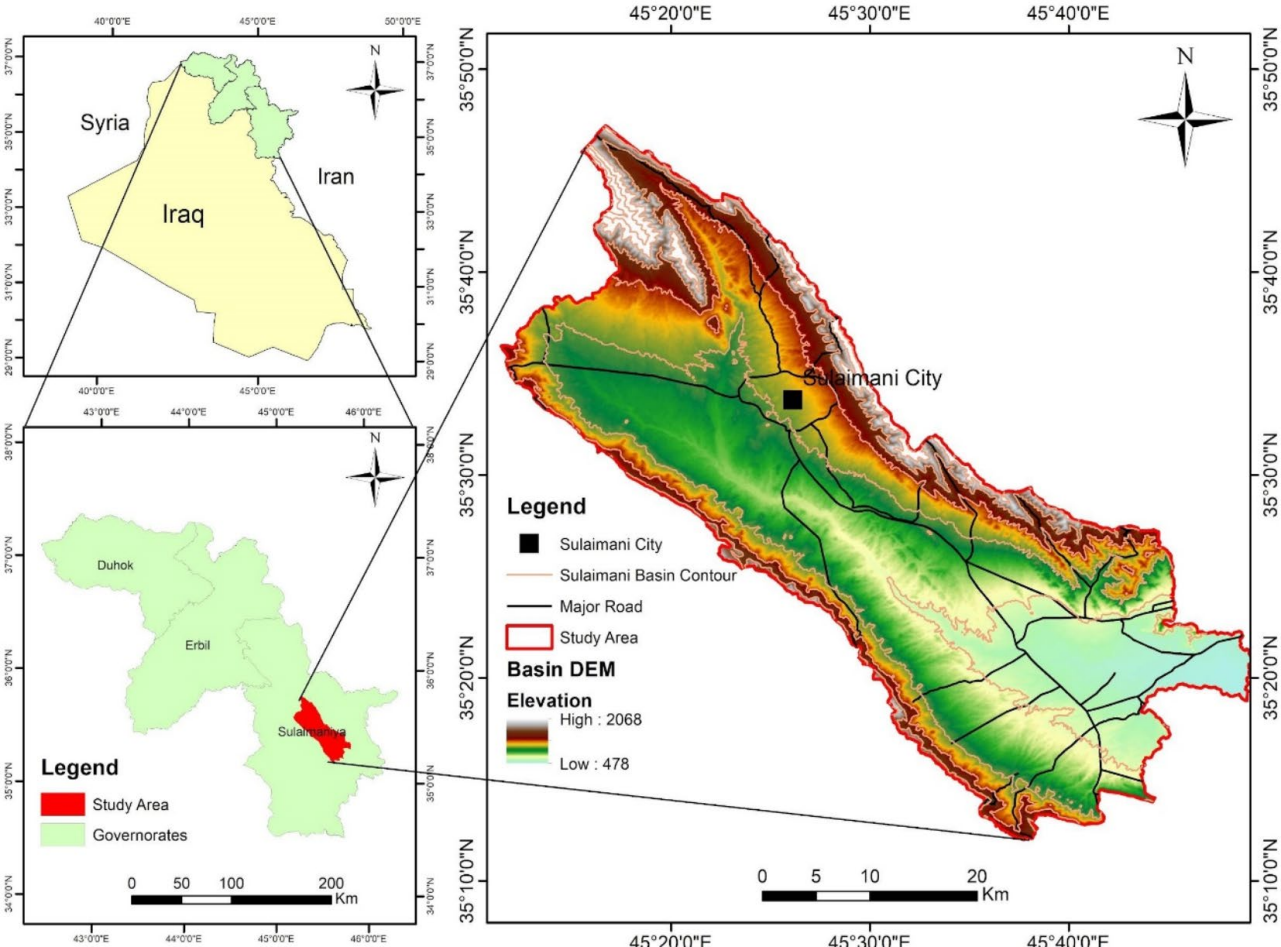
Display the location map of the study basin.

**Fig. 2:**
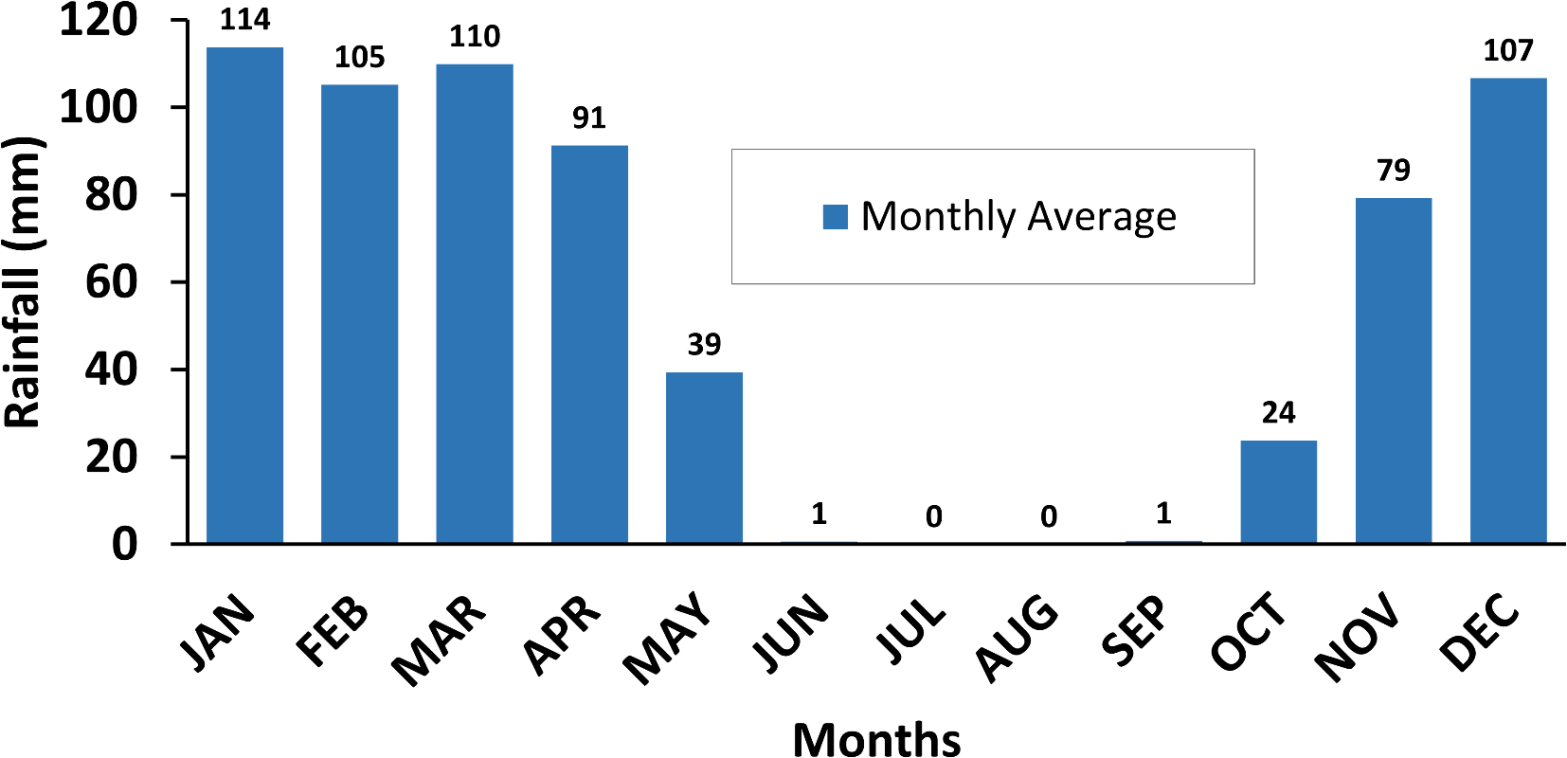
Statistical chart of Sulaymaniyah meteorological station 1978–2018.

The study area is located in the Sulaymaniyah Governorate, northeast of Iraq (Fig. 1). It covers an area of 1,303 km², with elevation ranging from 478 to 2,068 m above mean sea level (m.a.s.l.). The area is bordered by mountainous regions to the north, northeast, and southwest, while it flattens toward the southeast. The predominant climate in the SB is semi-arid, characterized by extreme cold and snowfall in winter, with temperatures ranging from − 2 to -10 °C, and hot summers, with temperatures ranging from 30 to 48 °C. The study area receives an average annual rainfall of 700 mm (Fig. 2), with January recording the highest monthly average rainfall while the minimum rainfall during the summer season^16,17^.

**Fig. 3:**
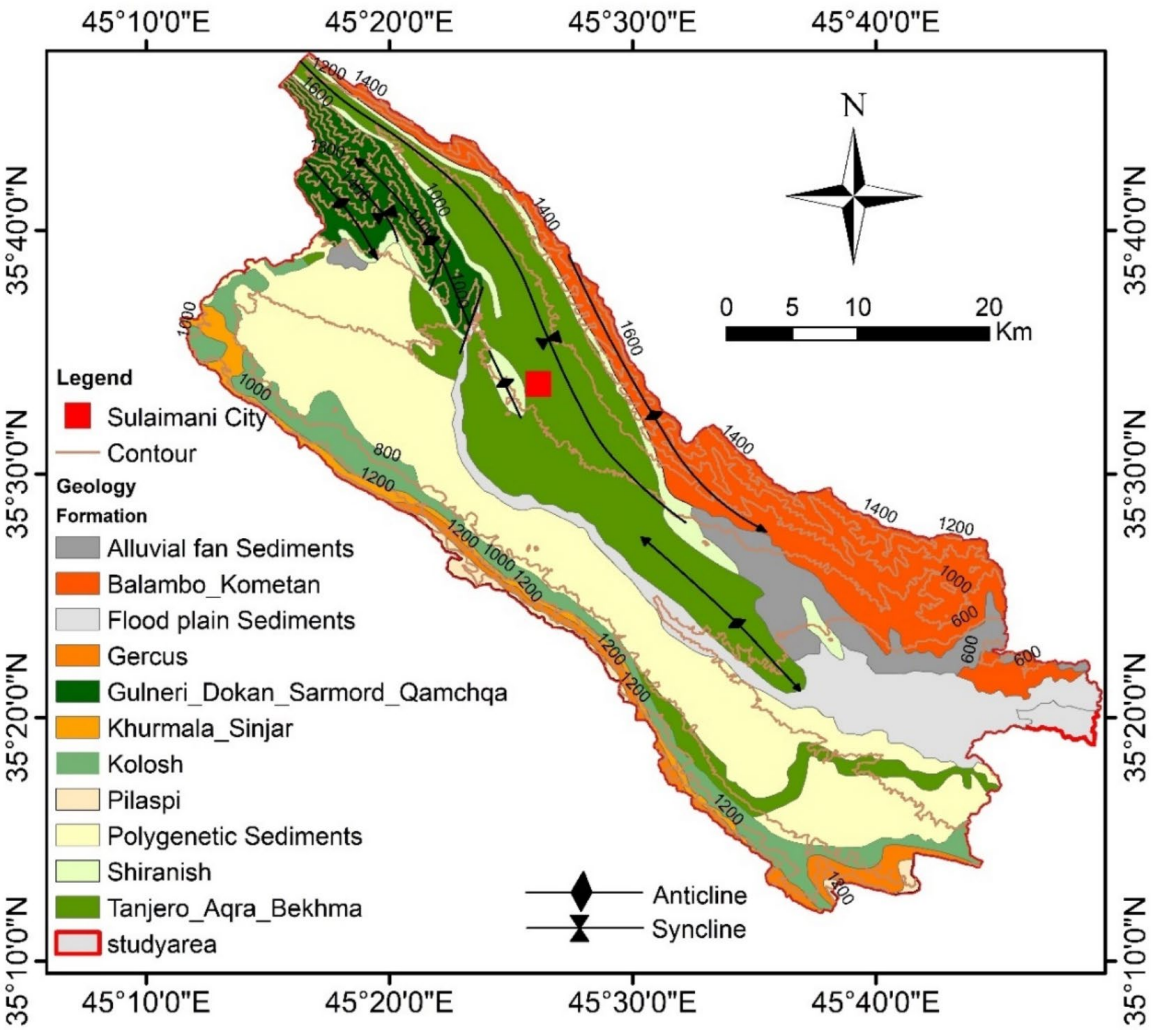
Lithology and tectonic structures of the study area^18^.

Geologically, the Sulaymaniyah Basin (SB) is characterized by a variety of lithostratigraphic units from the Cretaceous and Paleogene periods, including alluvial fan sediments, floodplain deposits, and polygenetic sediments. As illustrated in Fig. 3, the lithological sequence spans from older to younger formations: Qamchuqa, Kometan, Shiranish, Tanjero, Kolosh, Sinjar, Gercus, and Pilaspi^18^. From a hydrogeological perspective, the SB hosts diverse aquifer systems, including intergranular and karstic-fissured syatems. Previous classifications^19,20^ identified key aquifer systems within the basin: the karstic Bekhme aquifer, the fissured-karstic Pilaspi aquifer, intergranular aquifers, and complex aquifers^19^. further stratified the hydrogeological units as follows: Jurassic rocks forming Jurassic Karst Aquifers (JKA), Cretaceous rocks hosting Karstic-Fissured Aquifers (CKFA), fissured aquifers associated with the Cretaceous Qulqula Formation (CFA), intergranular aquifers within alluvial deposits (AIA), and the Tanjero Formation, which acts as an aquitard. These classifications highlight the basin’s complex groundwater system and its varied aquifer properties.

### Data and thematic layers Preparation

**Fig. 4:**
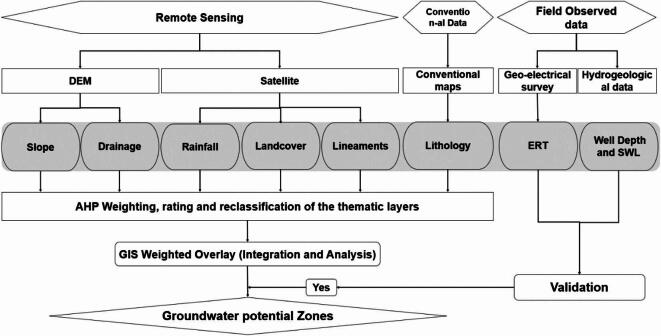
Conceptualized workflow of GWPZ.

A conceptual framework (Fig. 4) has been developed to delineate GWRPZ by incorporating key influencing factors including rainfall, geology, lineament density, slope, drainage density, and land use/land cover. These factors were selected based on their significant influence on groundwater dynamics (Table 1). The thematic layers were analyzed within a GIS environment using ArcGIS 10.8, with all data projected in the UTM coordinate system, Zone 38 N. Thematic layer preparation included digitizing lithological data from existing geological maps, extracting lineament features from remote sensing (RS) data through image processing in ArcGIS, and generating slope and drainage density maps using satellite-derived Digital Elevation Model (DEM) data. The LULC map was also derived from satellite imagery using image classification techniques. Hydrogeological data from groundwater wells, sourced from the Groundwater Directorate, were systematically classified and analyzed to validate the groundwater potential map.

**Table 1:** Thematic layers and source of data.

Thematic layers	Data	Resolution	Source
Geology	In Situ Data	1:250000	Iraqi Geological Survey
Lineament density	Satellite Image	30 m	USGS Earth Explorer, Landsat 8 Operational Land Imager (OLI)
Slope	DEM	12.5 m	ALOSPALSAR
Drainage density	DEM	12.5 m	ALOSPALSAR
LU/LC	Satellite Image	30 m	USGS Earth Explorer, Landsat 8 Operational Land Imager (OLI)
Precipitation	Satellite	Resample 150 m	CHIRPS Daily Dataset

### AHP

The AHP is multicriteria decision analysis framework developed by^5,21^, works based on pairwise comparison matrix (Table 2) used to derive the priority scale of the criterion relying on preferences using Saaty’s scale (Table 3). The AHP helps the decision makers to compare the relative importance of the complex decision making of various criteria to transfer into a single level decision making. The normalized weight for the criterion was assigned from the constructed comparison square matrix for rainfall, geology, slope, drainage density, LULC, and lineament criteria. Furthermore, the consistency ratio (CR) was determined from the normalized eigenvector to determine the suitability of the constructed matrix in which the CR ratio < 0.1 is acceptable, in contrast, the CR > 0.1 suggests that the initial comparison matrix needs to be revised. The normalized weights (Eq. 1) and CR are determined using the following steps respectively.

1 $$\:\text{W}\text{i}=\frac{{\text{G}}_{\text{i}}}{{\sum\:}_{\text{i}=1}^{\text{n}}{\text{G}}_{\text{i}}}$$

where Wi is the weight of criterion i using geometric mean, $\:{G}_{i\:\:\:}$is the principal eigenvector element for criterion i, and $\:{\sum\:}_{i}^{n}{G}_{i}\:\:$is the sum of the principal eigenvector elements for all criteria. Finally, CR of normalized weights was assessed using the (Eq. 2) as

2 $$\:\text{C}\text{R}=\frac{\text{C}\text{I}}{\text{R}\text{I}}\:\:$$

Where the consistency index (CI) is expressed in Eq. (3) while RI is the random consistency index according to number of the criteria can be chosen.

3 $$\:\text{C}\text{I}=\frac{{\uplambda\:}\text{m}\text{a}\text{x}-\text{n}}{\text{n}-1}$$

λmax is the maximum eigenvalue derived from Eq. (4) where the matrix is (X), n is the matrix size.

4 $$\:\lambda\:max=\frac{1}{n}{\sum\:}_{i-1}^{n}\frac{\left(Xw\right)i}{wi}$$

Then the thematic layers were normalized to enable integration of all thematic layers into a weighted overlay analysis within the GIS environment, resulting in the final groundwater potential map.

After assigning the normalized weights of each criterion (Table 4) then the prepared thematic layers are integrated into the GIS environment to create a groundwater potential zone using weighted overlay analysis (Fig. 4). Further, (Eq. 5) the combination weighted linear method by assigning weights to each factor GWPZ model is generated.

5 $$\:\text{G}\text{W}\text{P}\text{Z}={\sum\:}_{\text{j}=1}^{\text{m}}\text{*}{\sum\:}_{\text{i}=1}^{\text{n}}\left(\text{w}\text{j}\text{r}\text{i}\right)$$

Where: GWPZ: Groundwater potential zone, *W*_*i*_ and *r*_*i*_: is weight of J theme and rank of *i* class, *m*: The total number of theme layers, *n*: The total number of feature classes.

**Table 2:** Pair-wise comparison matrix with normalized principal eigenvector.

Matrix	Geology	Lineament	Slope	Drainage density	LULC	Rainfall	normalized principal Eigenvector %
	1	2	3	4	5	6	
Geology	1	2	3	3	4	1	27.60
Lineament Density	1/2	1	3	5	3	1/2	20.36
Slope	1/3	1/3	1	3	2	1/3	10.52
Drainage density	1/3	1/5	1/3	1	1/2	1/4	5.26
LULC	1/4	1/3	1/2	2	1	1/5	6.75
Rainfall	1	2	3	4	5	1	29.52

**Table 3:** Scale of Saaty’s AHP^5^.

Description	Less influential	Equally influential	More influential
Extreme importance	1/9		
V. Strong Importance	1/7		
Strong Importance	1/5		
Moderate importance	1/3		
Equal importance		1	
Moderate important			3
Strong importance			5
V. strong importance			7
Extreme importance			9

**Table 4:** Weight of the thematic layers and feature classes.

Theme Criteria	Sub-Criteria Classes	Descriptive term	Assigned Rank	Normalized class Weight%	Normalized Theme Weights %
Rainfall [mm]	600–625	Moderate	3	9.8	29.5
	625–650	Moderate	3	11	
	650–675	Good	2	14.2	
	675–700	Good	2	19.7	
	700–725	Very good	1	20	
	725–750	Very good	1	25.3	
Geology	Alluvial fan sediment	Very good	1	43.8	27.6
	Floodplain Sediment	Very good	1	43.8	
	Polygenetic sediment	Very good	1	43.8	
	Qamchuqa	Moderate	3	18.7	
	Balambo- Komitan	Good	2	26.3	
	Shiranish	Poor	4	6.7	
	Tanjero- Aqra	Poor	4	6.7	
	Kolosh	V Poor	5	4.5	
	Sinjar	Good	2	26.3	
	Gercus	V Poor	5	4.5	
	Pilaspi	Good	2	26.3	
Lineament density Km/Km^2^	0-0.5	V Poor	5	4.9	20.4
	0.51-1	Poor	4	10.4	
	1.1–1.5	Moderate	3	16.7	
	1.6-2	Good	2	30.8	
	2.1-5	V Good	1	37.2	
Slope [Degree]	0–5	V Good	1	45.1	10.5
	5.1–10	Good	2	23.7	
	11–15	Moderate	3	18.4	
	16–25	Poor	4	8.3	
	26–69	V Poor	5	4.5	
Drainage density Km/Km^2^	0-0.5	V Good	1	40.4	5.3
	0.51-1	Good	2	34.1	
	1.1–1.5	Moderate	3	13.9	
	1.6–1.7	Poor	4	11.7	
LU/LC	Cropland	Good	2	26.3	6.8
	Forest	V Good	1	28	
	Grassland	Moderate	3	16.5	
	Shrubland	Poor	4	13.2	
	Wetland	V Poor	5	9.5	
	Water	V Poor	5	9.5	
	Impervious surface	Extremely poor	6	3.8	
	Bare land	Extremely poor	7	2.7	

### Validation

#### Hydrogeological data

The validation process is crucial for ensuring the accuracy and reliability of models. Among the various methods for validating groundwater models, hydrogeological and geophysical surveys are widely used^14,22–24^. In this study, two common approaches were employed to analyze and interpret the results of the AHP groundwater potential zones (GWPZ). The first approach involves the interpolation of hydrogeological well data, such as the static water level (SWL) at various depths and the overall well depth, to assess the correspondence between GWPZ results and actual groundwater conditions. Additionally, the evaluation of the model accuracy involves determining the correlation between the AHP model result and the well data, using area under the curve (AUC) and receiver operating characteristic (ROC).

#### Geoelectrical data

ERT is employed to assess and validate the efficacy of GIS-based GWPZ. ERT involves applying a constant direct current into the ground through two current electrodes and measuring the resulting voltage at two potential electrodes^25,26^. This method utilizes a multielectrode and multi-cable system spread on the ground, with the positions of current and potential electrodes dependent on the chosen electrode array geometry. Data from six profiles within the SB were gathered utilizing a Wenner-Schlumberger configuration. The Wenner-Schlumberger array provides good vertical resolution and reasonable horizontal resolution^15,25^. Notably, two survey profiles featured an electrode spacing of 10 m with a profile length of 720 m, while four ERT profiles had an electrode spacing of 5 m, spanning a profile length of 360 m. The software “RES2DINV” version 4.8.10 facilitated 2D inversion analysis and interpretation. RES2DINV was iteratively employed to invert field data up to five times, ensuring that absolute error values (ABS) remained below 5%.

## Result and discussion

### AHP model

#### Geology

**Fig. 5:**
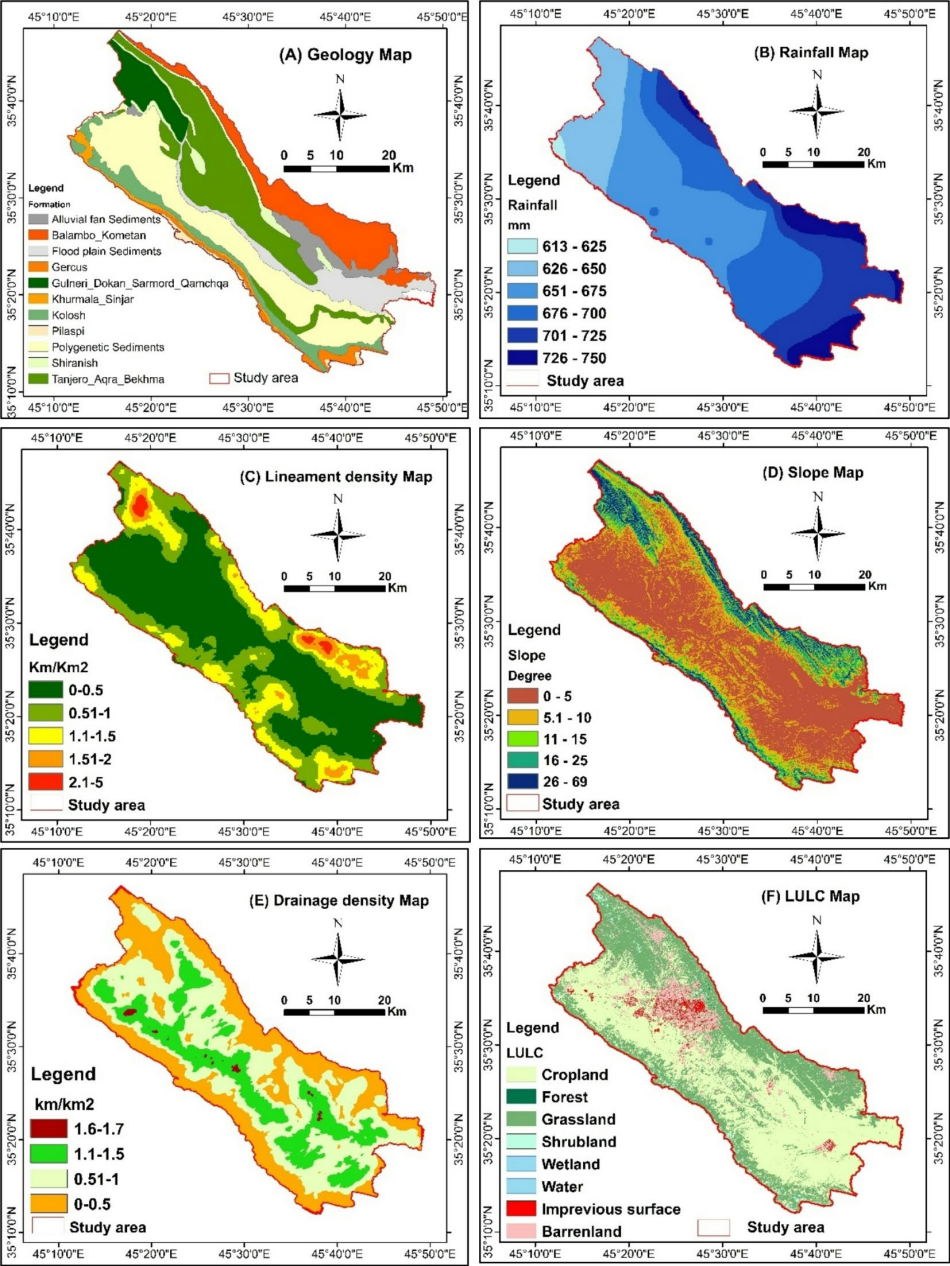
Reclassified thematic layers (**a**) Geology, (**b**) rainfall, (**c**) Lineament density, (**d**) Slope, (**e**) Drainage density, (**f**) LU/LC.

The occurrence and distribution of groundwater are primarily determined by geology^23,27,28^. The pairwise comparison results indicate that geology is the second most significant parameter, with a normalized weight of 0.276. The SB region is covered by various lithological units, including Alluvial Fan Sediment, Floodplain Sediment, Polygenetic Sediment, Qamchuqa, Balambo, Kometan, Shiranish, Tanjero-Aqra, Kolosh, Sinjar, Jercus, and Pilaspi (Fig. 5a). The most favorable for groundwater potential is the intergranular unit, with a weight of 43.8%, covering 15.7% of the total basin area. The fissured unit, with a normalized weight of 26.3%, ranks second and covers 25% of the SB. The karstic unit, classified as moderate potential, has a weight of 18.7% and covers 11% of the basin area. The aquitard unit is classified as having poor potential, with a normalized weight of 6.7%, covering 45% of the SB. The aquiclude unit, with very poor potential, has a normalized weight of 4.5% and covers 45% of the total basin area. Previous hydrogeological studies in the SB have identified the same aquifer units^17,19,20^.

#### Rainfall

**Fig. 6:**
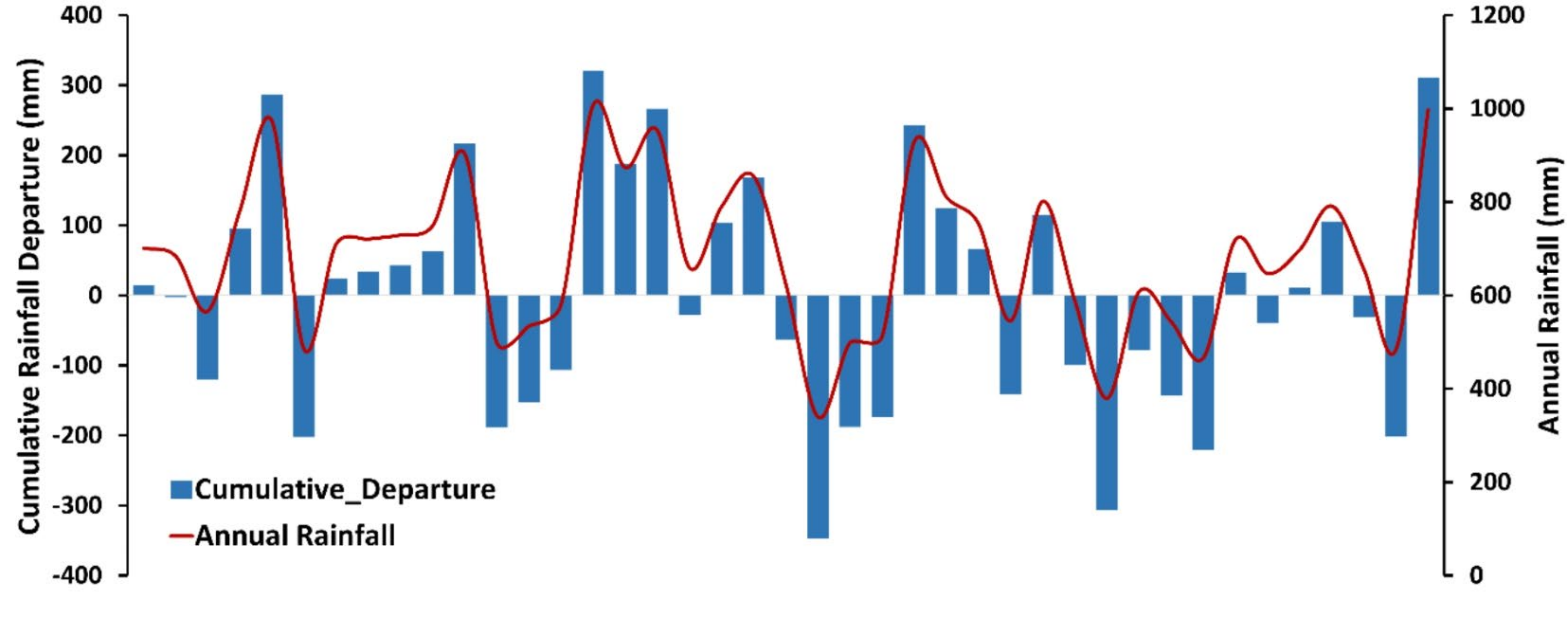
Cumulative rainfall departure for the period (1978–2018).

Climate plays a crucial role in the hydrological cycle, with rainfall being the primary source of groundwater recharge^27,29^. Initially, rainfall data collected for the period 1978–2018 was obtained to explore the relationship between rainfall and groundwater recharge using cumulative rainfall departure (CRD) (Fig. 6). Positive CRD values indicate years with above-average rainfall (wet conditions) suggests a higher potential for groundwater recharge in the area, whereas negative values denote years with below-average rainfall (dry conditions). The results reveal that fluctuations in rainfall departure generally correlate with the amount of rainfall. During periods of above-average rainfall, the area experiences increased infiltration, whereas below-average rainfall leads to drawdown in the static water level (SWL) due to reduced rainfall^17,19,30^.

In the AHP analysis, the rainfall was identified as the most significant contributing factor, with a normalized weight of 0.295. Accordingly, the rainfall data was categorized into six classes (Fig. 5b) based on precipitation records from 2010 to 2020. These classes range from those with the least influence on groundwater to those with the greatest influence: Class 1 (600–625 mm, rank = 6), Class 2 (626–650 mm, rank = 5), Class 3 (651–675 mm, rank = 4), Class 4 (676–700 mm, rank = 3), Class 5 (701–725 mm, rank = 2), and Class 6 (725–750 mm, rank = 1). Although the differences between rainfall classes are not substantial, climate change has significantly impacted rainfall patterns in the SB region. The eastern part of the region receives the highest rainfall, with a gradual decrease towards the western and northwestern parts of SB. Numerous studies have similarly identified rainfall as the primary factor contributing to groundwater availability^31–34^. Moreover, rainfall intensity significantly affects groundwater distribution and recharge. High-intensity rainfall over a short period tends to result in minimal percolation and increased surface runoff, whereas prolonged low-intensity rainfall leads to higher percolation rates with minimal surface runoff^25,28,29,35^.

#### Lineament density map

Lineaments significantly influence groundwater flow and infiltration^27,29,35,36^. The lineament map is depicted in Fig. 5c. The lineament parameter is the third most influential factor, with a normalized weight of 0.2036 (Table 4). The predominant lineament subclasses are classified as follows: very poor potential (0–0.5 km/km²), poor potential (0.6–1.0 km/km²), moderate potential (1.1–1.5 km/km²), good potential (1.6–2.0 km/km²), and very good potential (2.1–5 km/km²). In the SB region, lineaments generally align with the area’s main tectonic framework, trending in the NW-SE direction. Coarse lineaments are observed in the northern and northeastern parts of the study area, as well as in the southern and southwestern parts. These lineament patterns indicate potential recharge zones in higher elevations, with corresponding discharge areas in lower elevations, particularly in the central part of the study area, which exhibits higher groundwater potential. The occurrence and recharge of groundwater are closely associated with lineaments, which typically represent zones of fractures and cracks^14,22,37^.

#### Slope

Slope, representing the change in elevation, is a crucial factor in identifying potential groundwater zones. The slope gradient directly influences rainfall infiltration, a key source of groundwater recharge. The average slope gradient in the study region is 7%, as depicted in Fig. 5d. The AHP analysis assigned a weight of 0.105 to the slope factor, which was then reclassified into five categories based on their significance in groundwater recharge potential (Table 4). These slope categories are as follows: 0–5° (very good potential), 5.1–10° (good potential), 11–15° (moderate potential), 16–25° (low potential), and 26–69° (very low potential). The reclassified slope map indicated that areas with higher slopes are assigned lower weights compared to areas with lower slopes. The majority of the study area is characterized by low to moderate slopes, extending from the northwest to the southeast in the central part of SB, while higher slopes are found along the northern and northeastern borders. In the SB, the most favorable groundwater zones are found in low slope areas within the central region. This finding is consistent with previous studies^28^, which suggest that slope can be used to determine regional fluctuations in GWPZs.

#### Drainage density map

Drainage density is defined by the frequency and density of drainage features, measured in kilometers per square kilometer^38^. It is influenced by regional structure, geomorphology, and lithology. This parameter is ranked 6th among the influencing factors, with a calculated weight of 0.053 (Table 4). The drainage density was classified into four categories, as illustrated in Fig. 5e: the first class (0–0.5 km/km²) is attributed to low potential; the second class (0.51–1.0 km/km²) corresponds to medium potential; the third class (1.1–1.5 km/km²) is associated with high potential; and the fourth class (1.6–1.7 km/km²) is attributed to very high potential areas. According to^39^, high-permeability areas are often found in zones with low drainage density. In the SB region, higher drainage density is observed in the central part, extending from the northwest to the southwest, where high groundwater potential is indicated by low slope areas. The course stream, originating from the northwestern part of SB, flows across the basin toward the southwestern part.

#### LULC

The SB region has undergone significant changes in land utilization in recent decades, yet LULC remains a useful indicator for GWPZs. Land use and its areal extent affect groundwater recharge, with land covered by forests, water bodies, and agricultural areas generally enhancing groundwater recharge, while impervious surfaces like buildings and roads hinder infiltration^40–42^. In this study, the class weights of the LULC are cropland (26.3%), forest (28%), grassland (16.8%), shrubland (13.2%), wetland (9.5%), water body (9.5%), impervious surfaces (3.8%), and bare land (2.7%). Accordingly, LULC categories are ranked from high to low potential for groundwater recharge, as shown in Fig. 5f. High groundwater potential zones are associated with cropland. This finding is consistent with previous research^43^, which suggests that agricultural areas generally reduce surface runoff and enhance percolation rates^28^. In contrast, bare land and impervious surfaces contribute to medium to low groundwater potential zones, particularly in the northwestern part of the study area. Thus, rapid urbanization and human activities on hydrogeomorphology system threaten the existing groundwater recharge mechanism^44^.

#### Groundwater potential zones (GWPZs)

**Fig. 7:**
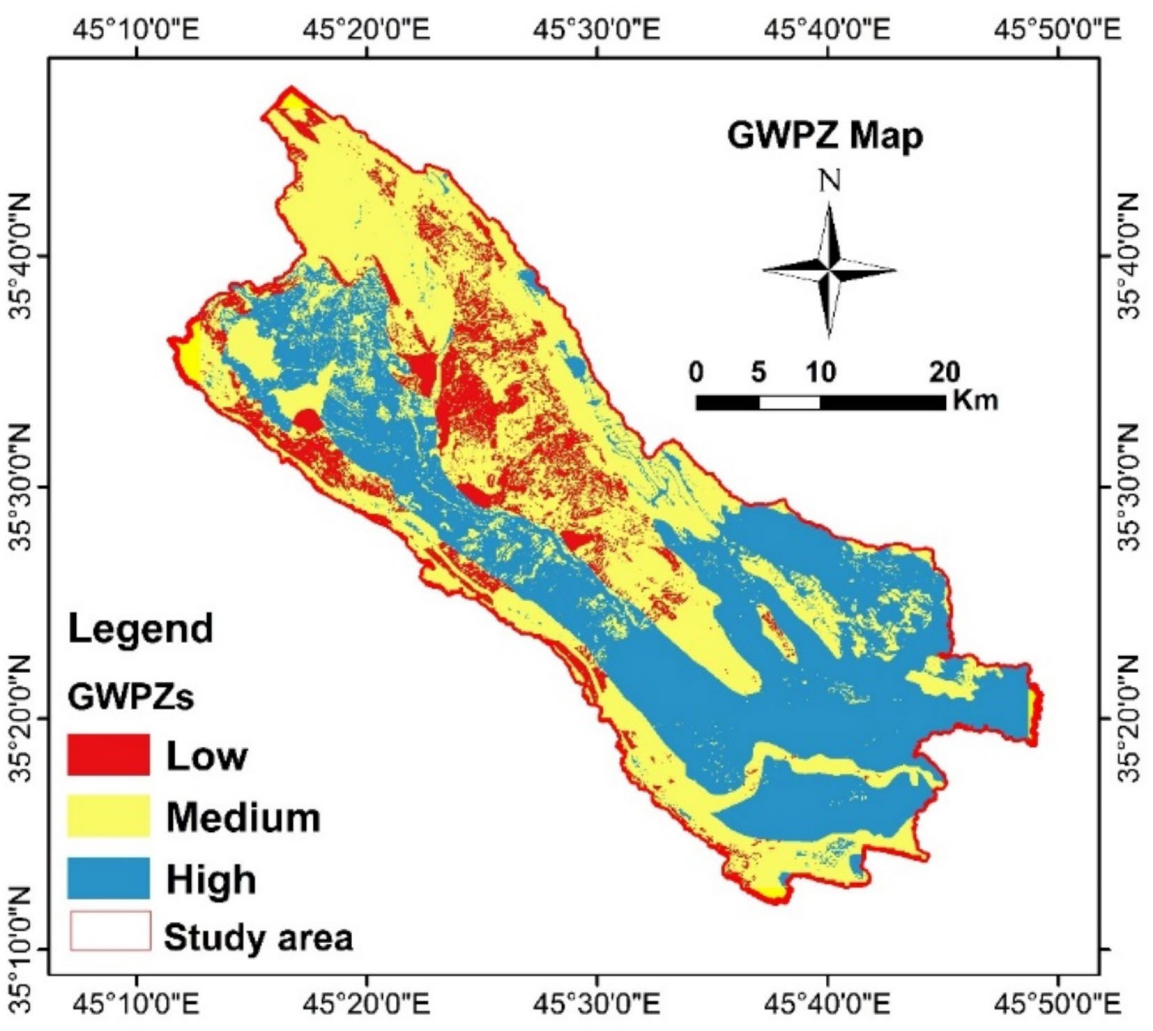
Groundwater prospective zones.

The six thematic maps were integrated to delineate the GWPZs. The results were categorized into three levels: low, medium, and high groundwater potential zones, with coverage areas of 147 km², 593 km², and 563 km², respectively (Fig. 7). High groundwater recharge potential is primarily located in the central region of the study area, extending from the northwestern to the southeastern part. This high potential is attributed to the geological presence of intergranular units, including floodplain (15.27%) and alluvial fan (0.43%) sediments. Additionally, increased rainfall contributes to larger recharge areas, particularly in low slope zones characterized by cropland and low drainage density^28,39^. Moderate groundwater potential zones are found in the northern to northwestern parts of the SB. This is influenced by the lithological units, including the Kolosh and Tanjero formations, which are classified as aquitard and aquiclude hydrogeological units^17,19^. Additionally, the moderate potential in SB is associated with low drainage density and high slope areas. Low groundwater potential zones are mainly affected by geology, including lower rainfall areas, impervious surfaces, and high slope regions^3,35,38,43^. The high groundwater potential areas as affected by high rainfall, intergranular hydrogeological units, low drainage density, and low slope. Conversely, low groundwater potential areas are characterized by high slopes and unfavorable land use conditions^3,14,28,29,35,39,45^.

### Accuracy of the AHP model

#### Hydrogeological data

**Fig. 8:**
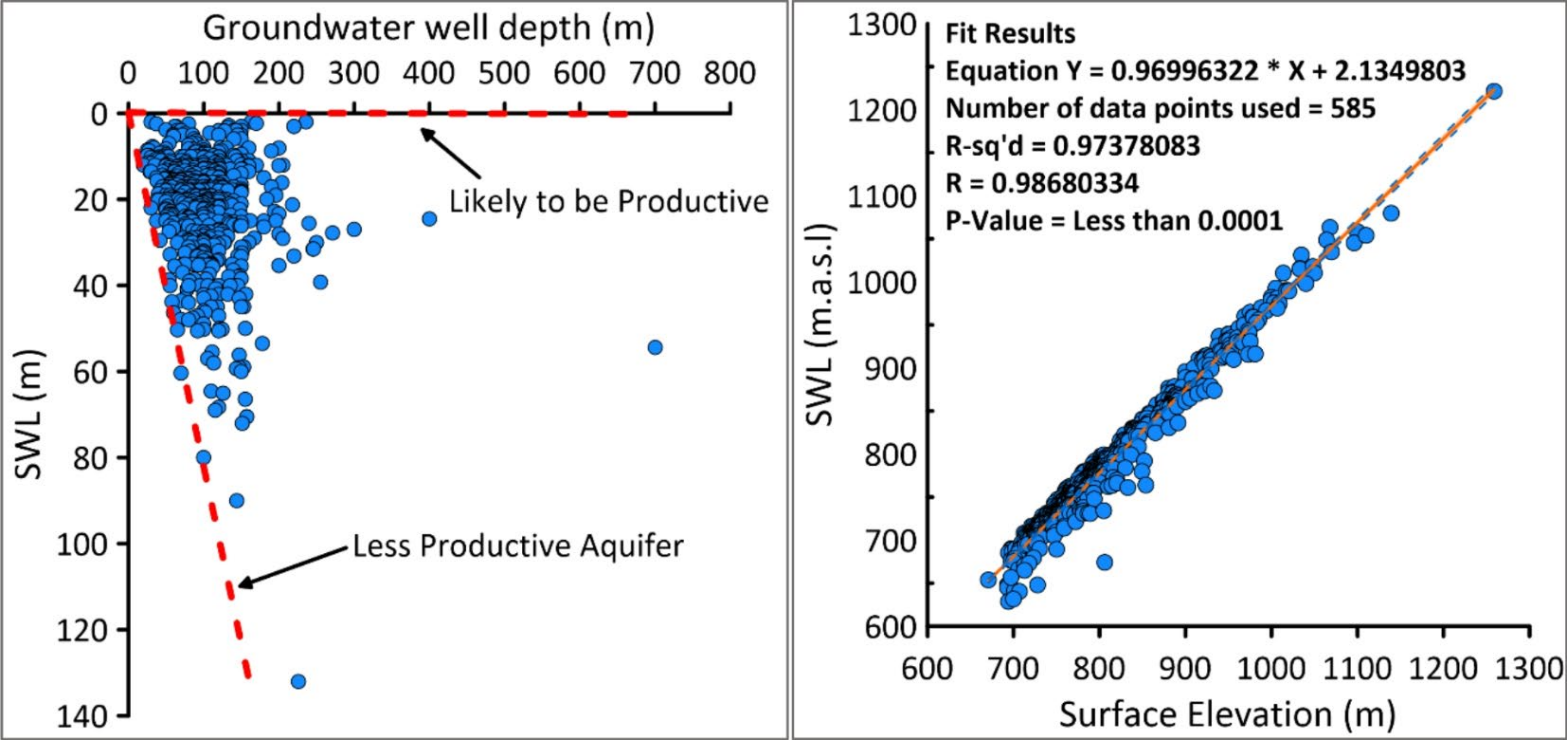
(**a**) Spatial distribution of depth to static water versus groundwater well depth, (**b**) Correlation between SWL (m a. s. l) and surface elevation (m).

**Fig. 9:**
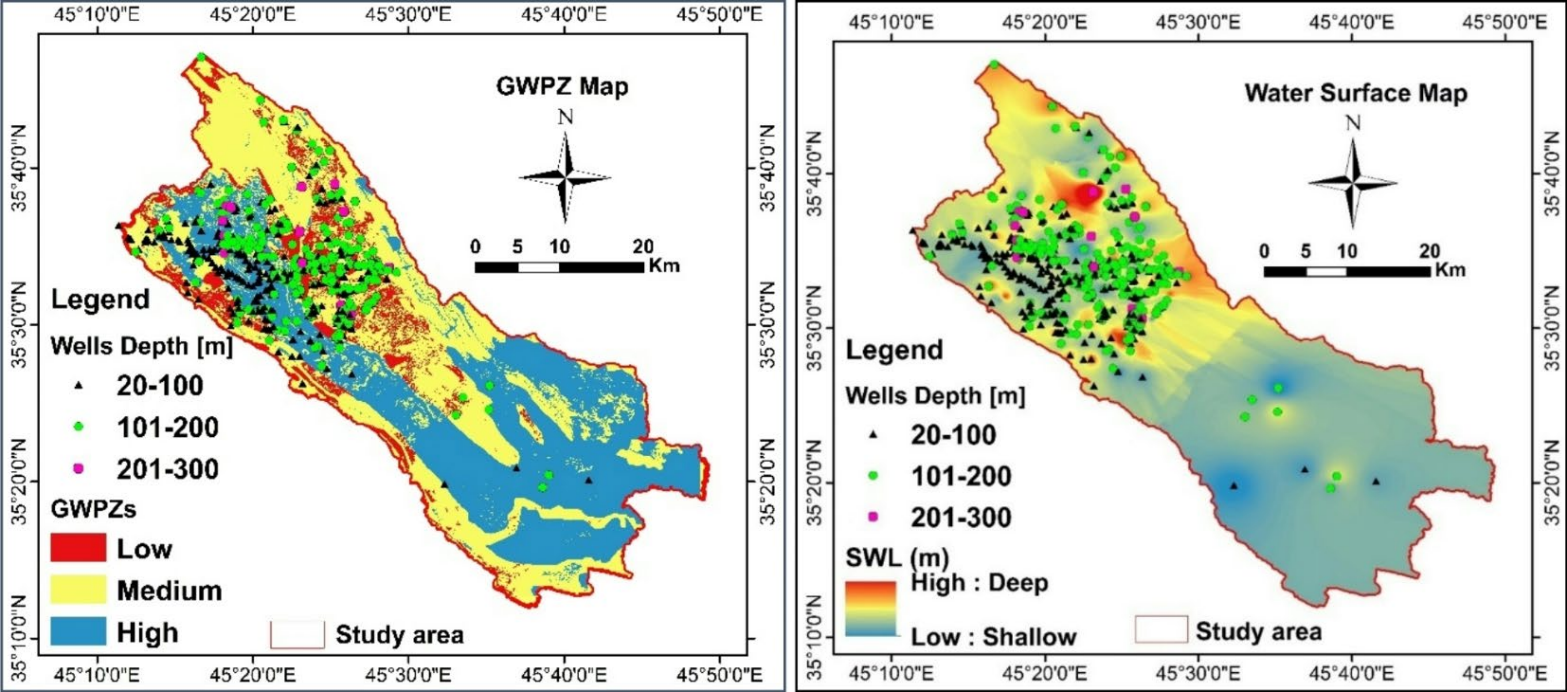
(*a*) Distribution of the existing wells in the groundwater potential zones. (**b**) Spatial interpolation of SWL and classified production well depths.

The robustness of the AHP model in this study was validated using hydrogeological and cross validation methods incorporating data from 536 wells. Firstly, spatial variations in static water level and well depths were analyzed (Fig. 8a and b), revealing strong correlation between SWL, well depth, and surface elevation. Figure 8a illustrates the spatial distribution and relation between SWL and well depth, it shows a clustered points in a specified well depths range between 20 and 200 m and SWL extending from 10 to 60 m depth. The dashed red line demonstrated two distinct borders between a less productive aquifer with a SWL > 60 m of SWL depth and a productive aquifer among the wells where the depth of the SWL close to SWL = 0. The result of linear regression analysis between surface elevation (m.a.s.l.) and SWL (m.a.s.l.), yielded an R-Squared value of 0.97, and a correlation coefficient (R) of 0.98 indicating a gravitationally driven aquifer system. Furthermore, wells were classified into three different classes based on their depth (Table 5). These classes are class 1 (< 100 m), class 2 (100–200 m), class 3 (200–300 m). Figure 9a shows most of the wells which placed in high GWPZs is in the first and second class with low depth (0–100 m), and stands 29% of the total number of the wells. The second (101–200 m) with 49% of the total wells placed in the moderate potential areas. The third class (201–300 m) are placed in moderate and low potential areas.

Geostatistical interpolation of SWL (Fig. 9b) confirms that areas with higher surface elevation have deeper SWL while the shallow SWLs observed in the middle part to the southeastern part of the SB. These findings align with the multi-aquifer system and lithological variations (Fig. 8a and b), where quaternary deposits exhibited high groundwater potential due to aquifer heterogeneity. The spatial distribution of SWL and well depths strongly supported the GWPZ model, with additional verification through six electrical resistivity tomography lines in underrepresented areas. This comprehensive validation demonstrated a strong agreement between observed data and the AHP-derived GWPZ model, confirming its reliability.

**Table 5:** Groundwater possibility zones and validation wells data.

GWPZs	Coverage area km^2^	Area %	Wells class (Depth)	Number of the wells	Wells %
High	563.438	43.23	< 100 m	155	29
Medium	593.2	45.51	100–200 m	263	49
Low	146.726	11.26	> 200 m	118	22
Sum	1303.364	100	-	536	100

**Fig. 10:**
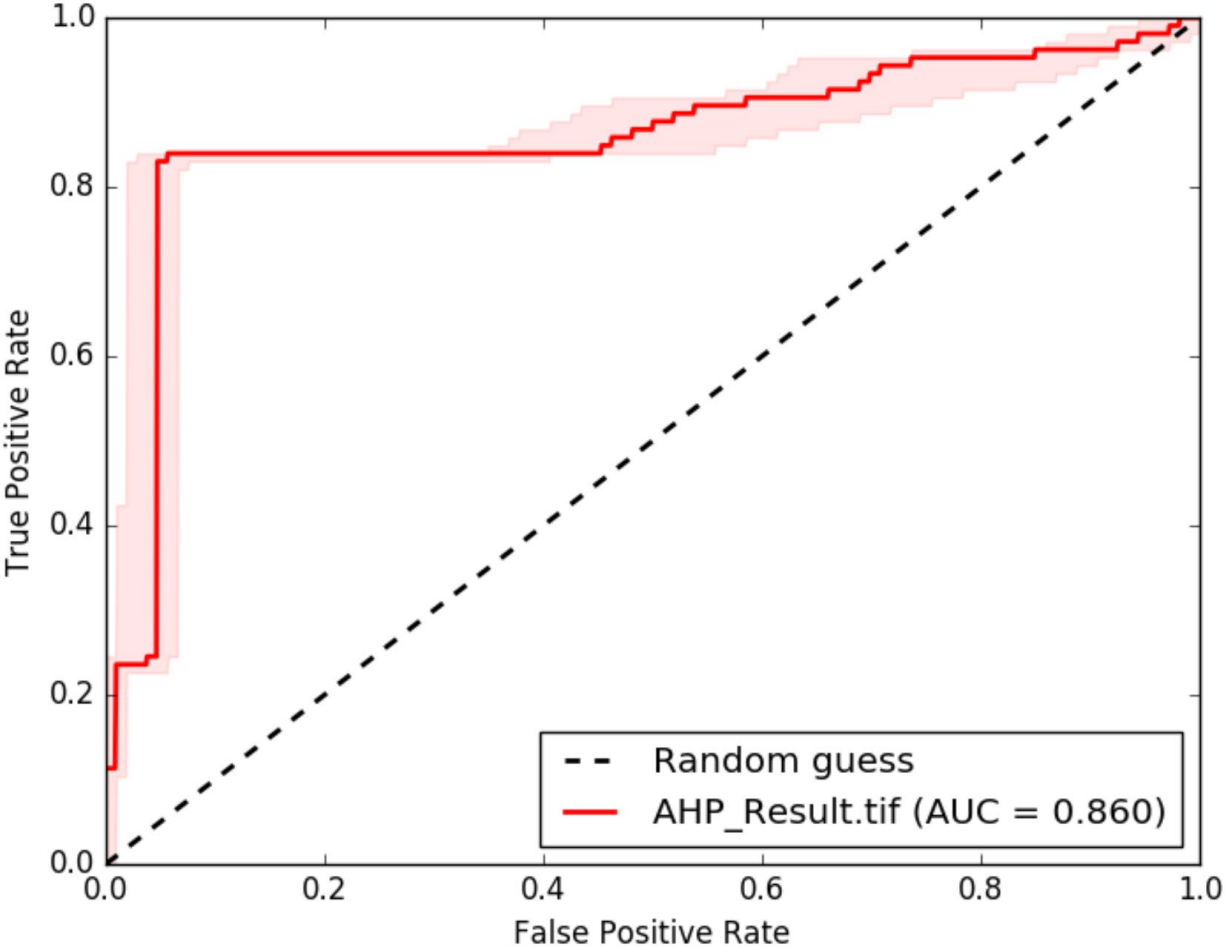
Validation result using ROC-AUC model.

Additionally, to determine the reliability and quantitative analysis of GWPZs the cross validation using ROC-AUC as shown in Fig. 10 were applied. The ROC curve demonstrated the cumulative percentage of the area along x-axis and water wells along y-axis which is a common statistics to determine the accuracy of the model classes^34,35,46–49^. AUC is common widely used statistic that counts how a parameters can be differentiates effectively between two classes^35,48^. The obtained AUC result of AHP model in GWPZ is 0.86 indicating that the AHP model approach produced a good accuracy model prediction.

#### Geoelectrical model

**Fig. 11:**
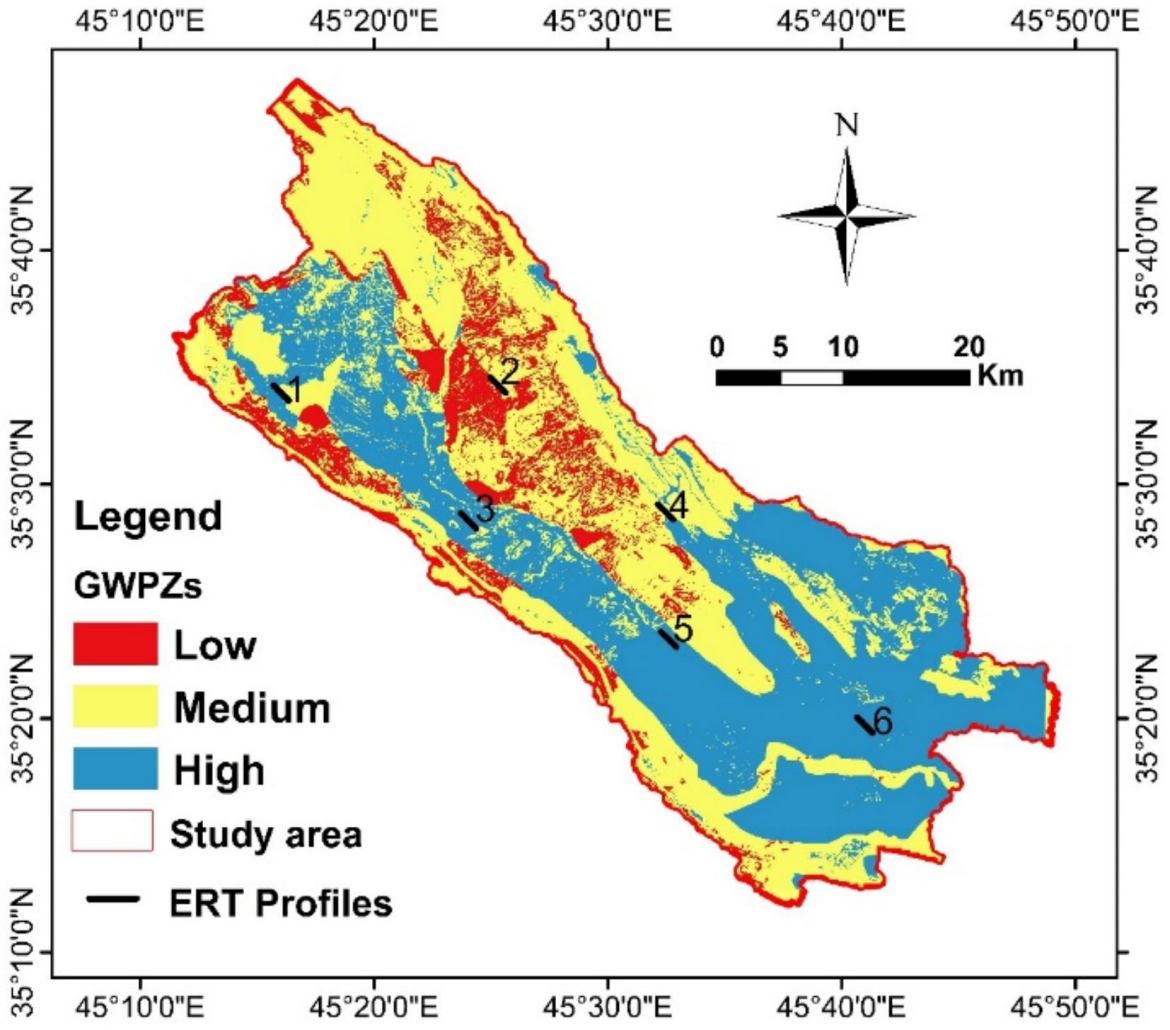
Shows distribution of ERT profiles.

**Fig. 12:**
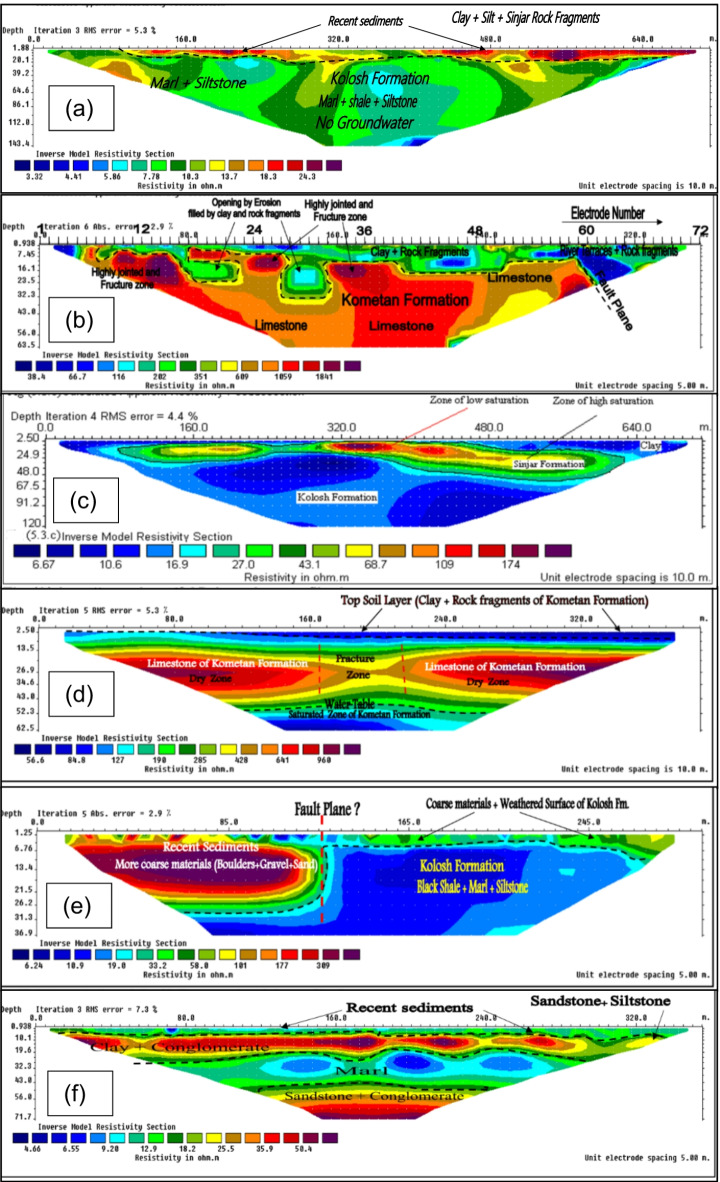
Resistivity inversion result of ERT profile.

To validate the delineation of groundwater potential zones (GWPZs) in the SB, six Electrical Resistivity Tomography (ERT) profiles were conducted using the Wenner-Schlumberger array (Fig. 11). The results from the first profile reveal a narrow resistivity range of 3–26 Ωm (Fig. 12a), distinguishing two zones. The upper zone consists of thin near-surface coarse materials, including clay, silt, and rock fragments from the Sinjar Formation, with moderate resistivity values ranging from 8 to 26 Ωm. The thickness of the Recent Sediments in this zone varies between 1 and 20 m. Beneath this, the Kolosh Formation is identified as the lower zone, characterized by resistivity values below 9 Ωm and extending to the bottom of the inverted section at a depth of approximately 143 m. This profile indicates a low-potential aquifer with limited groundwater storage capacity. The findings align with the GWPZ results for the same ERT profile, classifying the area as having medium to low groundwater potential, insufficient for sustaining water over long periods.

The second 2D profile (Fig. 12b) reveals a general topsoil layer with a thickness ranging from 1 to 16 m, primarily composed of clay interspersed with various sizes of rock fragments, which are weathered products of the Kometan Formation. Within this layer, two large openings in the Kometan Formation are identified, filled with clay and rock fragments. Below the topsoil, the limestone of the Kometan Formation is observed at depths ranging from 1 to 23 m, exhibiting resistivity values between 500 and 2800 Ωm. This layer, representing the lower part of the Kometan Formation, extends to the bottom of the inverted section at approximately 64 m depth. Based on these findings, the area does not appear promising for groundwater exploration, which is consistent with the GWPZs results indicating similarly low groundwater potential.

Profile 3 (Fig. 12c) highlights an elongated block of the Sinjar Formation, with resistivity ranging from 23 to 200 Ωm, forming a shallow aquifer. Notably, resistivity values between 20 and 120 Ωm at depths of 28–56 m indicate an excellent aquifer, both at this location and in other areas within the basin. The interfaces between the Sinjar block, surrounding clay, and Kolosh Formation are clearly delineated on the inverse section. Interestingly, the Kolosh Formation exhibits slightly higher resistivity (normally below 10 Ωm) due to the influence of the shallow, high-resistivity Sinjar Formation. A thin clay layer, approximately 2.5 m thick, overlies the aquifer beneath electrode 34, with thickness increasing to 14 m and 25 m beneath electrodes 9 and 63, respectively, in the NW and SE directions. The aquifer’s thickness ranges from 5 m beneath electrode 25 to 33 m beneath electrode 56. A high-resistivity zone (above 150 Ωm) observed beneath electrodes 32 to 38 likely corresponds to a dolomitization zone within the Sinjar Formation, where zones of low and high saturation are distinguishable by lower and higher resistivity, respectively. These findings provide promising insights for groundwater exploration, aligning with results from the GWPZs.

Profile 4 (Fig. 12d) reveals three distinct zones. The first is a low-resistivity layer (35–80 Ωm) consisting of clay, silt, and varying sizes of limestone fragments from the weathered surface. This topsoil layer has a thickness of 0–5 m, gradually increasing toward the southeastern part of the area. The second layer exhibits high resistivity (80–1200 Ωm), representing the dry zone of the Kometan Formation, detected at depths ranging from the surface to about 5 m and extending to the bottom of the inverse section at 64 m. A fracture zone with low resistivity is observed between electrodes 17 and 22, likely due to the presence of clay or marl within the fractures. Below a depth of 50 m, the resistivity of the Kometan Formation decreases to 40–80 Ωm, indicating the presence of groundwater. The ERT results suggest this site is suitable for groundwater exploration at specific depths, while the GWPZs results indicate a range of potential, predominantly moderate, across the area.

Profile 5 (Fig. 12e) reveals two distinct zones. The first zone, characterized by high resistivity (19–500 Ωm), has a thickness ranging from 4 m beneath electrode 35 in the northwest to approximately 30 m below electrode 20 in the southeast. This variability in resistivity is attributed to a mix of lithological components, including clay, silt, sand, gravel, and boulders. The second zone corresponds to the Kolosh Formation, identified by its low resistivity (6–18 Ωm) and composed of black shale, marl, and siltstone. This layer underlies the entire area and is exposed at the surface between electrodes 27 and 60, while at depths of 25–27 m beneath electrodes 1 to 27, possibly due to a fault near electrode 27. This profile lies within high to moderate groundwater potential zones; however, the ERT results emphasize the need for further groundwater investigations, including the assessment of existing wells in the vicinity.

The interpretation of Profile 6 (Fig. 12f) reveals distinct subsurface layers. The first layer, with a resistivity range of 5–15 Ωm, represents the topsoil, primarily composed of clay, with a thickness of approximately 5 m. Beneath it lies a sandstone and conglomerate layer with a resistivity of 20–60 Ωm and a thickness of about 15 m, indicative of an aquifer. Below this aquifer, another clay-dominated marl layer appears, with resistivity values of 5–15 Ωm and a thickness of around 20 m. The final layer, extending deeper, is another sandstone and conglomerate unit with resistivity ranging from 20 to 60 Ωm and a thickness of approximately 25 m. This profile clearly delineates the layers with water storage potential, confirming high groundwater potential in the area as supported by the GWPZs analysis.

The interpretation of all 2D electrical resistivity tomography (ERT) profiles demonstrates a strong agreement with the groundwater potential zones (GWPZs) identified in the study area, effectively validating the reliability and accuracy of the groundwater potential map. The ERT results successfully delineated subsurface features, including aquifer zones, low-resistivity clay and marl layers, and high-resistivity fractured or consolidated formations, which align closely with the groundwater potential classifications derived from the GWPZ model. This correlation underscores the model’s robustness in identifying zones with varying groundwater storage and flow potential. The validated GWPZs provide a scientifically sound basis for planning future groundwater exploration and sustainable management strategies, ensuring that high-potential zones are effectively targeted while minimizing exploration risks in less favorable areas.

### Implication for climate change

One of the most important pillars of water resources management and development is groundwater exploration. Recently, due to climate change and variation in rainfall pattern different issues related to water resources have been addressed. In this frame the United Nation (UN) recognized 17 sustainable development goals (SDGs), targeting minimizing the level of poverty, providing peace, and initiate sustainable environmental pillars for the future^50^. Particularly SDG6 aims to provide a sustainable water resources development and sanitation everywhere^51^. Since many previous researches highlighted the groundwater decline noticeably due to the rainfall variations^50,52,53^. Sustainable groundwater resources are further endangered by the detected decrease in rainfall and rise in temperature beside irrigation purposes which become a leading factor in populated areas. Moreover, there is a noticeable declining groundwater level in the area due to increasing of production rate associated to population, agriculture and industry beside variation in rainfall pattern^17,38^, These changes cause improve the current problems of decreasing groundwater level and tend to make a step harder to reach SDGs. However, there are associate constraints of insufficient data, and budgetary problems become the main issue for the researchers. Hence this study focused on RS and GIS environment as a cost-time effective techniques in groundwater management. Generally, the result of groundwater potential mapping shows a quite promising area to find groundwater, it provides vital information to different stake holders in SB.

## Conclusions

This study aimed to delineate groundwater potential zones using geospatial modeling and multi-criteria decision analysis via the analytical hierarchy process, supported by hydrogeological and geophysical investigations. The study successfully fulfilled the objectives, and the conclusions can be summarized as follows:

- Six thematic layers including rainfall, lithology, lineament density, slope, drainage density, and land use/land cover were integrated to identify GWPZs. Based on the assigned weights and ranks, the study area was classified into three zones: low (11.26%), moderate (45.51%), and high potential (43.23%). The high groundwater potential zones were primarily located in the central region, extending from the northwest to the southwest. Moderate to low potential areas were predominantly found in the northern and northwestern parts, influenced by geological features and land cover. These areas, characterized by fractures, also facilitate groundwater recharge.
- The GWPZ map was validated using geophysical surveys and existing hydrogeological well data. Results showed that 29% of wells in the high-potential zone had depths less than 100 m, 49% in the moderate-potential zone had depths between 100 and 200 m, and 22% in the low-potential zone were deeper than 300 m. Additionally, electrical resistivity tomography (ERT) results closely matched the GWPZ classifications, demonstrating ERT’s effectiveness as a tool for verifying groundwater potential sites.
- While this study successfully identified groundwater potential sites, data limitations constrained the model’s accuracy. Future research should incorporate additional hydrogeological variables, such as depth-to-groundwater and recharge rates, for improved accuracy and robustness. A multidisciplinary approach combining advanced geophysical techniques, geospatial tools, and hydrogeological data is recommended for more precise GWPZ mapping.
- The findings of this study provide critical insights into regional groundwater resource management. The approach and results can support the development of effective policies and sustainable groundwater supply strategies, contributing to the achievement of the United Nations Sustainable Development Goal 6 (Clean Water and Sanitation).

## Data Availability

The datasets used during this research were obtained with permission from the Sulaymaniyah Groundwater Directorate and are available from the corresponding author upon reasonable request.
